# A comparison of mosquito densities, weather and infection rates of Aedes aegypti during the first epidemics of Chikungunya (2014) and Zika (2016) in areas with and without vector control in Puerto Rico

**DOI:** 10.1111/mve.12338

**Published:** 2018-09-17

**Authors:** R. Barrera, M. Amador, V. Acevedo, M. Beltran, J. L. Muñoz

**Affiliations:** ^1^ Entomology and Ecology Team, Dengue Branch Centers for Disease Control and Prevention San Juan Puerto Rico; ^2^ Molecular Diagnostic Laboratory, Dengue Branch Centers for Disease Control and Prevention San Juan Puerto Rico

**Keywords:** Aedes aegypti, Chikungunya, dengue, mosquito control, Zika

## Abstract

In Puerto Rico, the first records of the transmission of Chikungunya (CHIKV) and Zika (ZIKV) viruses were confirmed in May 2014 and December 2015, respectively. Transmission of CHIKV peaked in September 2014, whereas that of ZIKV peaked in August 2016. The emergence of these mosquito‐transmitted arboviruses in the context of a lack of human population immunity allowed observations of whether the outbreaks were associated with Aedes aegypti (Diptera: Culicidae) densities and weather. Mosquito density was monitored weekly in four communities using sentinel autocidal gravid ovitraps (AGO traps) during 2016 in order to provide data to be compared with the findings of a previous study carried out during the 2014 CHIKV epidemic. Findings in two communities protected against *Ae. aegypti* using mass AGO trapping (three traps per house in most houses) were compared with those in two nearby communities without vector control. Mosquito pools were collected to detect viral RNA of ZIKV, CHIKV and dengue virus. In areas without vector control, mosquito densities and rates of ZIKV detection in 2016 were significantly higher, similarly to those observed for CHIKV in 2014. The density of *Ae. aegypti* in treated sites was less than two females/trap/week, which is similar to the putative adult female threshold for CHIKV transmission. No significant differences in mosquito density or infection rates with ZIKV and CHIKV at the same sites between years were observed. Although 2016 was significantly wetter, mosquito densities were similar.

## Introduction

The incessant global expansion of the dengue viruses (DENVs 1–4) has challenged public health capacities for decades [World Health Organization (WHO), 2012; Bhatt *et al*., 2013; Messina *et al*., 2016]. The recent geographical expansions of Chikungunya virus (CHIKV) (Wahid *et al*., 2017) and Zika virus (ZIKV) (Gubler *et al*., 2017), which have transmission cycles similar to those of the DENVs, demonstrates that prevailing approaches to preventing dengue are not working and human populations may be at risk for infection by other enzootic arboviruses that may eventually emerge in urban areas (Braack *et al*., 2018). The co‐circulation and co‐infection of humans with DENV, CHIKV and ZIKV have already been reported (Carrillo‐Hernandez *et al*., 2018). The main vector of DENV, CHIKV and ZIKV in urban epidemics in tropical and subtropical areas is the domestic mosquito *Aedes aegypti* [Centers for Disease Control and Prevention , 2018].

The best strategy for the prevention of epidemics of these arboviruses, other than the use of effective and safe vaccines, is to maintain *Ae. aegypti* densities below the minimum number of mosquitoes required for local transmission, or below a mosquito density threshold (Focks *et al*., 2000). The policy of waiting for a declaration of a dengue epidemic in order to implement *Ae. aegypti* control has not worked well in the past. Modelling studies suggest that an introduced arbovirus can cause an outbreak if the number of mosquitoes exceeds a threshold or critical level and, in turn, the number of mosquitoes required to cause an outbreak depends on herd immunity, ambient temperature and the frequency of virus introductions (Focks *et al*., 2000). Generally, fewer mosquitoes are required if herd immunity is low or non‐existent, temperature is high, and viruses are frequently introduced. The introduction of CHIKV and ZIKV into naïve human populations in the Americas provides an opportunity to learn more about the mosquito density threshold required to prevent rampant outbreaks. The DENVs have been circulating in the Americas for decades and are hyperendemic in many countries (Gubler, 2011) and hence it is more difficult to establish such thresholds as populations have varying levels of herd immunity. A density threshold that is established according to newly circulating arboviruses is expected to represent a conservative estimation of the maximum mosquito densities required to prevent DENV outbreaks in endemic areas because the densities of *Ae. aegypti* necessary to cause outbreaks are likely to be higher.

Thresholds to prevent urban yellow fever epidemics were established in the past and have been recommended as reference data in strategies to prevent DENV transmission (Brown, 1977). However, a number of studies have reported DENV transmission in contexts in which the Breteau index was below the threshold of five out of 100 houses with at least one container with immature stages of *Ae. aegypti* (Bowman *et al*., 2014). Studies in Cuba and Taiwan suggested DENV transmission took place when Breteau indices were close to one (of 100 houses) (Sanchez *et al*., 2006; Chang *et al*., 2015). Unfortunately, indices of the prevalence of immature stages in containers found in houses (e.g. the house, Breteau and container indices) do not generally correlate well with adult mosquito abundances (Focks, 2003). Additionally, *Ae. aegypti* uses cryptic aquatic habitats in houses (e.g. septic tanks, inundated roofs and basements, drains) (Barrera *et al*., 2008, Pilger *et al*., 2011), as well as in public areas (e.g. storm drains, trash in abandoned lots) (Paploski *et al*., 2016). Missing important aquatic habitats of *Ae. aegypti* could lead to underestimations of mosquito prevalence and provide a false sense of security. Monitoring adult densities of *Ae. aegypti* has been difficult in the past because of a lack of practical traps to deal with the complex anthropophilic behaviour of this mosquito (Barrera, 2016). Newer traps, such as the electro‐mechanic BG‐Sentinel trap (Krockel *et al*., 2006) or passive gravid traps, such as the CDC autocidal gravid ovitrap (AGO trap) (Mackay *et al*., 2013) or the gravid *Aedes* trap (Eiras *et al*., 2014) allow the establishment of programmes for the surveillance of *Ae. aegypti* based on female adults that transmit viruses. Capturing adult *Ae. aegypti* using these devices also allows for testing for the presence of arboviruses. The presence of arboviruses in local *Ae. aegypti* females is indicative of the nearby presence of infected humans as this mosquito does not move far during its lifetime (Reiter, 2007) and serves as a proxy for human infections or xenomonitoring (Barrera *et al*., 2017).

The invasions of CHIKV and ZIKV in immunologically naïve human populations also provide opportunities to investigate possible differences in transmission dynamics, such as differences in vector densities and other environmental parameters. Comparisons of epidemics of dengue and Zika concluded that reproduction numbers were similar when data for the same localities were compared, but differed in comparisons of epidemics of the same virus in other localities. Variations in environmental conditions are more important than differences between viruses (Funk *et al*., 2016). Similarly, a comparison of Chikungunya and Zika epidemics suggested similar transmission potential in the same areas (Riou *et al*., 2017). Variations among localities in the importance of weather parameters, such as temperature and precipitation, and their potential impact on both vectors and viruses have been reported (He *et al*., 2017; Lourenço *et al*., 2017; Riou *et al*., 2017). Other variables that are important to consider in the dynamics of these epidemics, but perhaps more difficult to evaluate, are differences in the vector competence of *Ae. aegypti* populations in different geographical settings (Zouache *et al*., 2014; Kauffman & Kramer, 2017).

A longitudinal study of the impact of mass trapping using AGO traps on the density of *Ae. aegypti* mosquitoes in southern Puerto Rico, begun in October 2011, provided an opportunity to document and compare mosquito densities and incidences of arboviruses in *Ae. aegypti* following the invasions of CHIKV in 2014 and ZIKV in 2016. The AGO trap is a black plastic container with a volume of approximately 19 L that contains 10 L of water and a small hay packet as an attractant, and a sticky glue board that captures gravid females of *Ae. aegypti* seeking a place to lay eggs (Mackay *et al*., 2013). Recently, the surveillance trap‐based presence of CHIKV in female *Ae. aegypti* and mosquito density were found to be 10 times higher in adjacent communities without AGO control traps than in communities with such traps (Barrera *et al*., 2017). An investigation into the prevalence of CHIKV antibodies in the residents of these communities revealed 50% protection in areas with mass trapping (Lorenzi *et al*., 2016). The aims of the present study were to elucidate whether *Ae. aegypti* densities were associated with ZIKV infection rates in female adult mosquitoes, how differences in weather parameters between years might influence mosquito densities and the presence of arboviruses, and whether natural infection rates of mosquitoes with CHIKV and ZIKV differed in the same locations with and without vector control.

## Materials and methods

### Study sites and treatments

Four communities were involved in this investigation, including two communities with vector control (La Margarita, Villodas) and two without vector control (Arboleda, La Playa). La Margarita (17°58′18″ N, 66°18′10″ W; 327 houses) is a neighbourhood in which vector control consisted of source reduction and larvicidal treatments applied at the beginning of a study conducted in December 2011, to which three AGO traps per house in over 80% of houses were subsequently added (Barrera *et al*., 2014a). In Villodas (17°58′13″ N, 66°10′48″ W; 241 houses), for the purposes of comparison, only source reduction and larvicidal treatments were applied in December 2011, but, from February 2013 onwards, the installation of three AGO traps per home in most homes was added to the treatment regime to determine if the number of mosquitoes declined to the low levels observed in La Margarita (Barrera *et al*., 2014b). In the present study, in order to compare mosquito densities in neighbourhoods without vector control, two nearby communities, Arboleda (17°58′46″ N, 66°17′23″ W; 398 houses) and Playa (17°57′59″ N, 66°18′10″ W; 269 houses) were also studied. La Margarita and Arboleda are middle‐income communities, whereas Playa and Villodas are less affluent and houses have bigger patios. No other mosquito control measures were applied in these areas during the study.

### Mosquito sampling and weather

Female *Ae. aegypti* sampling was conducted every week in the four study sites during January–December in 2014 and 2016. The AGO traps used for surveillance and control were similar, except that those used for control had a mesh funnel at the entrance of the trap to discourage lizards from entering the capture chamber (Acevedo *et al*., 2016). The number of surveillance AGO (SAGO) traps needed to cover a neighbourhood and the number of control traps per home had been determined previously (Mackay *et al*., 2013). The total number of traps used for control purposes depends on the number of householders consenting to participation. Coverage with control traps exceeded 80% in the two communities from the beginning of the study. In total, 44 sentinel SAGO traps and 793 AGO control traps were placed in La Margarita, 27 SAGO and 570 AGO control traps in Villodas, and 30 and 28 SAGO traps (no control traps) in Arboleda and Playa, respectively. The number of *Ae. aegypti* mosquitoes was recorded every week in each SAGO trap and both types of trap were serviced and the water, hay pack and sticky glue board replaced every 2 months. One meteorological station recording air temperature, rainfall and relative humidity (RH) (HOBO Data Logger; Onset Computer Corp., Bourne, MA, U.S.A.) was placed in the centre of each of La Margarita, Villodas and Arboleda to collect hourly data during January–December in both years. No meteorological station was placed in Playa because this community was only 200 m away from La Margarita and therefore the data provided by the station in La Margarita were used for both communities.

### Detection of DENV, CHIKV and ZIKV in mosquitoes

Mosquito pools (1–20 female *Ae. aegypti*) collected from SAGO traps every week in all four locations during 2014 and 2016 were prepared. During 2014, detection of only DENV and CHIKV RNA was attempted following adapted reverse transcription polymerase chain reaction (RT‐PCR) protocols because ZIKV had not yet been detected in the Americas (Lanciotti *et al*., 2007; Santiago *et al*., 2013; Barrera *et al*., 2017). During 2016, a trioplex RT‐PCR was used to simultaneously detect RNA of DENV, CHIKV and ZIKV (Barrera *et al*., 2018; Santiago *et al*., 2018). Viral RNA of several arboviruses, including DENV, CHIKV and ZIKV, can be detected in dried mosquitoes at room temperature or on sticky surfaces exposed in the field for more than 1 week (Bangs *et al*., 2001; Mavale *et al*., 2012; Burkhalter & Savage, 2017).

### Statistical analyses

Generalized estimating equations (GEEs) were used to test the null hypotheses of no statistical differences in the density of *Ae. aegypti* females/trap/week between years (2014, 2016), between sites (two untreated, two treated), and their interaction (years*sites). The following weather variables were also included in the analysis: accumulated rainfall (2 and 3 weeks before sampling), and average daily temperature and RH (during the 3 weeks before sampling). Data for accumulated rainfall in the second and third weeks before sampling were used because rainfall within 7 days of sampling should not have any impact on the number of adult mosquitoes captured other than indirect effects mediated by RH on adult survival. For temperature and RH, the average of each during the previous 3 weeks was used as these variables may influence the longevity and survival of adult mosquitoes. The test used a negative binomial distribution of mosquito abundance, log link and autoregression order one for repeated measures. Changes in weather variables (weekly rainfall, average daily temperature or RH) between years, sites and their interaction were evaluated using a generalized linear model (GLM) with a normal distribution and identity link. For the latter tests (GLM), untransformed weather variables describing weekly (rainfall) or daily (temperature, RH) values were used. PooledInfRate Version 4.0 was used to calculate and compare virus infection rates (IRs) or, more properly, viral RNA detection from mosquito pools (Biggerstaff, 2016). Infection rates were reported as per 1000 mosquitoes. Any pair of IRs (e.g. CHIKV vs. ZIKV IR per site) was considered not significantly different (α = 0.05) if the confidence interval of the difference included zero. A vector index (VI) (Jones *et al*., 2011) was used to calculate the expected number of infected mosquitoes/trap/week as the product of the proportion of infected mosquitoes multiplied by the average mosquito density multiplied by 1000.

## Results

**Figure 1:**
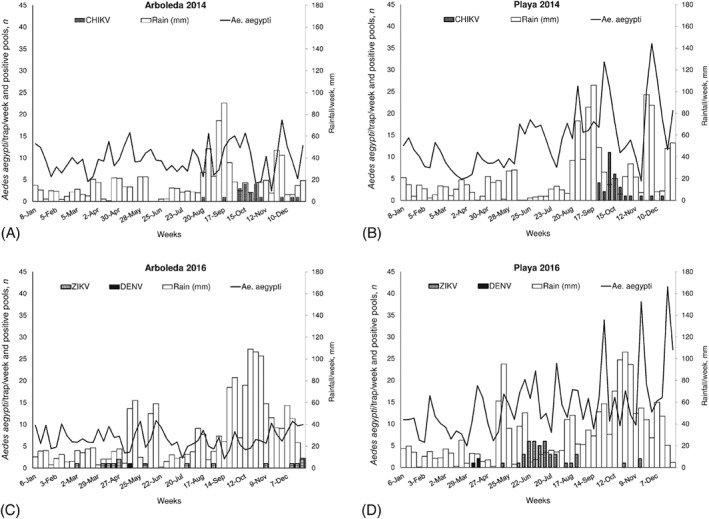
Comparisons of average numbers of female *Aedes aegypti*/trap/week, numbers of mosquito pools/week positive for the presence of viral RNA of Chikungunya (CHIKV), dengue (DENV) or Zika (ZIKV) viruses, and accumulated rainfall during the third and second weeks before sampling between years (2014, 2016) in two communities without vector control in southern Puerto Rico: (A) Arboleda in 2014, (B) Playa in 2014, (C) Arboleda in 2016, and (D) Playa in 2016.

**Figure 2:**
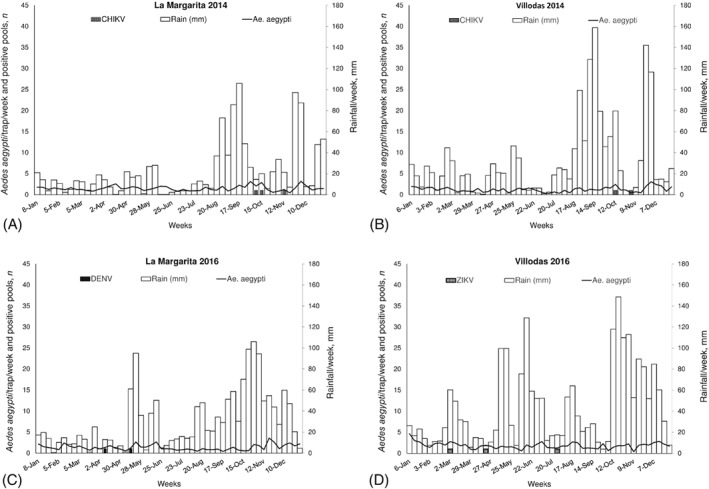
Comparisons of average numbers of female *Aedes aegypti*/trap/week, numbers of mosquito pools/week positive for the presence of viral RNA of Chikungunya (CHIKV), dengue (DENV) or Zika (ZIKV) viruses, and accumulated rainfall during the third and second weeks before sampling between years (2014, 2016) in two communities with vector control in southern Puerto Rico: (A) La Margarita in 2014, (B) Villodas in 2014, (C) La Margarita in 2016, and (D) Villodas in 2016.

In total, 6591 and 6585 trap‐week mosquito samples were collected across all four sites in 2014 and 2016, respectively. The total number of female *Ae. aegypti* collected was 40 220 in 2014 and 37 242 in 2016. The GEE analyses did not detect significant differences between years in *Ae. aegypti* density (Wald χ^2^ = 0.29; *P* > 0.05) or RH (χ^2^ = 3.6; *P* = 0.058), but did find significant effects of site (χ^2^ = 577.1; *P* < 0.001), the interaction years*sites (χ^2^ = 46.8; *P* < 0.001), rainfall (Wald χ^2^ = 0.29; *P* > 0.05) and temperature (χ^2^ = 7.6; *P* < 0.01). The interaction of years*sites was significant, indicating that the density of female *Ae. aegypti* in Arboleda was lower in 2016 than in 2014, but similar at the other three sites in 2014 and 2016 (Table 1, Figs 1 and 2). Mosquito densities in untreated sites were 5.5–9.5 times greater than in treated sites (Table 1). Average densities in treated sites were below two females/trap/week throughout the study periods (Table 1).

**Table 1:** Weekly mean numbers of female *Aedes aegypti* per trap, weather variables, number of positive pools, infection rate (per 1000 mosquitoes) with Chikungunya (CHIKV), dengue (DENV) or Zika (ZIKV) viruses, and vector index (per 1000 mosquitoes) in 2014 and 2016 in communities with and without vector control in southern Puerto Rico.

	No vector control				Vector control			
	Playa		Arboleda		La Margarita		Villodas	
	2014	2016	2014	2016	2014	2016	2014	2016
*Ae. aegypti* females/trap/week								
Mean SE Total	13.89 0.38 20 019	14.53 0.38 21 093	9.75 0.18 15 043	6.74 0.13 10 492	1.51 0.04 3430	1.36 0.04 3101	1.24 0.05 1728	1.84 0.06 2556
Rainfall per week, mm								
Mean SE Total	21.98 3.52 1165	30.27 3.77 1574	15.95 2.39 846	29.64 3.96 1541	21.98 3.52 1165	30.27 3.77 1574	31.45 5.11 1667	43.17 5.16 2244
Daily temperature per week, °C								
Mean SE	27.15 0.17	27.40 0.17	26.25 0.18	26.22 0.16	27.15 0.17	27.40 0.17	26.86 0.18	27.04 0.18
Daily RH per week, %								
Mean SE	74.81 0.29	77.46 0.46	74.97 0.28	76.14 1.31	74.81 0.29	77.46 0.46	76.48 0.36	79.25 0.46
Pools positive for CHIKV, *n* Infection rate Vector index	31 2.483 43	0	19 2.463 24	0	3 1.754 3	0	2 2.129 3	0
Pools positive for ZIKV, *n* Infection rate Vector index	—	42 2.064 30	—	13 1.394 9	—	0	—	3 1.205 2
Pools positive for DENV, *n* Infection rate Vector index	0	3 0.145 2.1	0	1 0.098 0.7	0	2 0.670 0.9	0	0

RH, relative humidity; SE, standard error.

Accumulated rain during the second and third weeks before sampling significantly influenced mosquito density, although increases in mosquito density following rainfall in treated areas were small (Figs 1 and 2). The results of a GLM analysis comparing rainfall per week between years (χ^2^ = 14.0; *P* < 0.001) and sites (χ^2^ = 11.5; *P* < 0.001) were significant. The year 2016 was wetter than 2014 and the greatest rainfall occurred in Villodas in both years (Table 1, Figs 1 and 2). The interaction term years*sites was not significant (χ^2^ = 0.5; *P* > 0.05). Daily air temperature varied significantly among sites (χ^2^ = 37.6; *P* < 0.001), but not between years (χ^2^ = 0.9; *P* > 0.05) and the interaction term was not significant (χ^2^ = 0.7; *P* > 0.05). Daily RH varied significantly between years (χ^2^ = 18.4; *P* < 0.001), suggesting that 2016 was more humid than 2014 and reflecting more rainfall in 2016. Relative humidity also varied among sites (χ^2^ = 14.7; *P* < 0.001) and the interaction term was not significant (χ^2^ = 2.0; *P* > 0.05). Arboleda showed the lowest air temperature and Villodas demonstrated the highest RH in both years (Table 1). Rainfall recorded during the first part of the year was lower in 2014 than in 2016 at all three meteorological stations (Figs 1 and 2).

**Figure 3:**
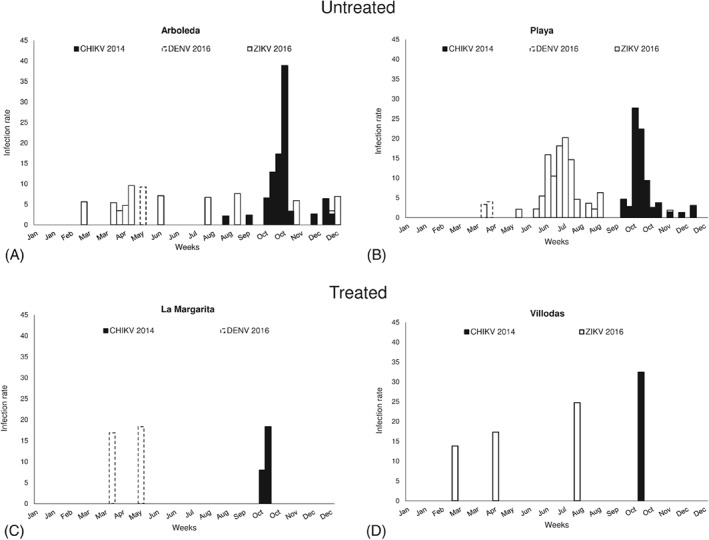
Infection rates (mosquitoes per 1000)/trap/week of *Aedes aegypti* with Chikungunya (CHIKV), dengue (DENV) or Zika (ZIKV) viruses in (A, B) two untreated and (C, D) two treated communities in southern Puerto Rico in 2014 and 2016: (A) Arboleda, (B) Playa, (C) La Margarita, and (D) Villodas.

In 2014, CHIKV‐positive pools of female *Ae. aegypti* numbered 50 in the untreated sites and five in the treated areas; no positive pools were found in 2016 (Table 1). The number of CHIKV‐positive pools was greatest in the neighbourhood with the highest mosquito density (Playa). There were no statistical differences between sites in overall CHIKV IR in samples taken during June–December in 2014 (1.75–2.48 infected mosquitoes/1000 mosquitoes; *P* > 0.05) (Table 1). Weekly IR varied widely, reaching maximums of 38, 18, 28 and 32 infected mosquitoes/trap/week/1000 mosquitoes in Arboleda, La Margarita, Playa and Villodas, respectively (Fig. 3). The average VI (expected number of infected mosquitoes/trap/week/1000 mosquitoes) was larger in both communities without vector control (24 in Arboleda and 43 in Playa) than in treated areas (3 in each of La Margarita and Villodas) (Table 1). Positive pools of *Ae. aegypti* females were detected in consecutive weeks at untreated sites in October 2014 (Figs 1 and 3). Some of the results of CHIKV infection in mosquitoes for these study sites have been presented previously (Barrera *et al*., 2017) and are shown here to facilitate comparisons with the 2016 ZIKV and DENV results.

During 2016, 58 pools across all four sites were ZIKV‐positive; 55 of these were sourced from untreated areas. The other three positive pools were found in Villodas and none were found in La Margarita (Table 1, Figs 1 and 2). With respect to CHIKV, the site with the greatest number of mosquitoes also had the largest number of ZIKV‐positive pools (Playa). Overall IRs did not differ significantly between sites (1.2–2.0 ZIKV‐infected mosquitoes/1000 mosquitoes; *P* > 0.05) (Table 1, Fig. 3). Weekly ZIKV IRs varied widely, reaching maximums of 10, 0, 20 and 25 infected mosquitoes/1000 mosquitoes/week in Arboleda, La Margarita, Playa and Villodas, respectively (Fig. 3). Overall IRs of *Ae. aegypti* with CHIKV in 2014 and ZIKV in 2016 did not differ significantly among sites (*P* > 0.05), with the exception of La Margarita, where no ZIKV‐positive pools were found. Average VIs were higher in untreated (9 in Arboleda and 30 in Playa) than in treated (0 in La Margarita and 2 in Villodas) sites (Table 1). In 2016, ZIKV was detected in mosquitoes as early as March in Arboleda and Villodas, and later in May, with more frequent detections during June and July, in Playa (Figs 1 and 3).

No DENV‐positive mosquito pools were found in 2014, but six were found in 2016. Four positive pools were identified in untreated areas and the other two in La Margarita, a treated site (Table 1).Overall mosquito IRs with DENV (0.15–0.67) were lower than those with CHIKV or ZIKV, but given the small number of occurrences, no statistical comparisons were performed (Table 1). Average VIs were also lower for DENV (0.7–2.1). Dengue virus was detected in April 2016 in Playa (three pools), in April and May 2016 in La Margarita (two pools), and in May 2016 in Arboleda (one pool) (Figs 1 and 2).

## Discussion

In the Americas, CHIKV has been circulating since 2013 (Zeller *et al*., 2016) and ZIKV since 2013 (Ayllon *et al*., 2017) or 2014 (Faria *et al*., 2017). Their rapid spread was possible because the region's population was immunologically naïve in urban areas with well‐established *Ae. aegypti* populations, which previously were transmitting the DENVs. Fewer mosquitoes are needed to cause local transmission of DENV when herd immunity in the human population is low (Focks *et al*., 2000). In the current study, the presence of ZIKV in *Ae. aegypti* during its emergence in Puerto Rico in 2016 was compared with the presence of CHIKV in 2014 (Barrera *et al*., 2017) in females of *Ae. aegypti* in four communities with different mosquito densities and levels of vector control. For both CHIKV in 2014 and ZIKV in 2016, mosquito densities and rates of virus detection were significantly higher in the two communities without vector control than in the treated communities. Average mosquito densities in communities with AGO traps were lower than two females/trap/week and only a few positive pools and no virologic evidence in mosquitoes of ongoing local transmission (lack of detection in several consecutive weeks) were found. A serologic study of CHIKV antibodies in residents of the four communities showed that the prevalence of immunoglobulin G (IgG) antibodies was at least 50% lower in people with AGO traps than in those in communities without traps (Lorenzi *et al*., 2016). A vector density threshold that seemed protective against CHIKV of less than three female *Ae. aegypti*/SAGO trap/week was proposed (Barrera *et al*., 2017, 2018). Mosquito densities collected in the same treated areas in 2016 in the present study were also below that threshold and a similarly low ZIKV incidence in *Ae. aegypti* was found. As DENVs have been circulating in Puerto Rico for decades and herd immunity is high (Arguello *et al*., 2015), the mosquito density threshold for local transmission may be higher than two or three females/SAGO trap/week. Thus, keeping *Ae. aegypti* density below this threshold may also protect against dengue outbreaks.

Weather conditions during 2016 seemed more favourable for mosquitoes and arboviral transmission than in 2014 because of the more abundant rainfall and higher humidity. Yet, contrastingly, the only change in mosquito density observed in 2016 occurred in one of the untreated communities (Arboleda), in which densities were lower than in 2014. Longterm data (since 2013) show that mosquito density in that particular location has decreased over time (data not shown). The results also seem to indicate that ZIKV detections in that location were less clustered in time (weeks) than CHIKV detections in 2014, when average mosquito density was 30% higher. Significant effects of rain and humidity on *Ae. aegypti* density through time have been observed previously in Puerto Rico (Barrera *et al*., 2011; Lega *et al*., 2017), but the significant increase in rainfall and humidity in 2016 did not translate into the higher vector densities expected.

The overall IR or fraction of pools of female *Ae. aegypti* positive for CHIKV or ZIKV did not differ significantly between years in the four communities, and there were no significant differences between treated and untreated sites for each virus. The lack of significant differences in IRs between viruses in the same locations is concordant with epidemiologic data indicating that the transmission potentials of *Ae. aegypti*‐borne arboviruses, such as DENV, CHIKV and ZIKV, are similar in the same locations (Funk *et al*., 2016; Riou *et al*., 2017). Although the results of the present local study cannot be extrapolated to the whole island, the finding of similar IRs for CHIKV and ZIKV may relate to the fact that similar proportions of people (20–25%) were infected with CHIKV in 2014 and ZIKV in 2016 in Puerto Rico (Hills *et al*., 2017). The finding of similar IRs across the four communities with and without vector control does not seem to indicate that IRs are reasonable indicators of risk because the density of *Ae. aegypti* was several times higher in untreated areas. It is suggested that a better indicator of risk may be the VI, which is derived by multiplying the proportion of infected mosquitoes/week by mosquito density/trap/week (Jones *et al*., 2011). Vector index values were several times higher in the two untreated areas, with values in the range of 20–40 expected infected mosquitoes (per 1000)/trap/week.

Comparing IRs in mosquitoes is difficult because of differences in sampling tools, number of samples, and analytic methodologies. Overall IRs of *Ae. aegypti* with CHIKV (7–12 per 1000) and ZIKV (5–19 per 1000) sampled using sentinel AGO traps for 2 weeks in other communities in Puerto Rico (CDC, unpublished data, 2018) were similar to those observed in the present study, although IRs derived from weekly samples at peak transmission were higher in this study (CHIKV, IR = 38; ZIKV, IR = 25). Previous studies have reported CHIKV IRs comparable with those observed here (4–32 per 1000) (Sang *et al*., 2008; Diaz‐Gonzalez *et al*., 2015; Dzul‐Manzanilla *et al*., 2015). Previous reports of ZIKV IRs in *Ae. aegypti* were higher than those observed in this investigation, such as in mosquitoes collected around patients in Mexico (53–172 per 1000) (Guerbois *et al*., 2016) and in mosquitoes collected in Senegal (71 per 1000) (Diallo *et al*., 2014). In the present study, DENV IRs were very low (0.15–0.67 per 1000), virus detections were infrequent, and no DENV was detected in 2014 in the same study sites. During the 2010 DENV epidemic in Puerto Rico, a higher IR (26 per 1000) in rural areas was observed (CDC, unpublished data, 2010) and was similar to those reported in Colombia (33 per 1000) (Perez‐Castro *et al*., 2016) and Venezuela (15–17 per 1000) (Urdaneta *et al*., 2005). A study of IRs in *Ae. aegypti* collected at 100 m around houses with positive or negative dengue cases in Thailand found 13 and 0.6 infected mosquitoes per 1000, respectively (Yoon *et al*., 2012).

It is interesting to note that although CHIKV and ZIKV were introduced into Puerto Rico at different times of year, their overall incidences in *Ae. aegypti* were similar. For example, CHIKV was confirmed in May 2014 (Sharp *et al*., 2016), which usually corresponds with the first peak in the rainy season in Puerto Rico (Barrera, 2010), but in 2014 that peak in rainfall was practically absent. Subsequently, CHIKV spread rapidly throughout the island and started to fade away after December 2014, when it is typically cooler and drier. The CHIKV epidemic peaked in September 2014 in Puerto Rico during the second and typically greater peak in rainfall. The timing of the epidemic peak coincided with the period during which most CHIKV‐positive pools were observed in the study sites (September–October 2014). Zika virus was first detected in December 2015 and the epidemic peaked in August 2016. This virus was detected early in the study sites (March) and most positive pools were identified during May–July 2016. Clearly, ZIKV had a longer period of circulation than CHIKV throughout 2016 before the advent of the cooler and drier season in December, which corresponds with the boreal winter at higher latitudes and is when most dengue epidemics usually wane (Barrera, 2010). However, it should be noted that ZIKV was able to gain a foothold and eventually spread during the drier and cooler season (December 2015 to March 2016). Zika outbreaks in French Polynesia, Colombia and the State of Bahia, Brazil were reported to have taken off during a relatively dry season (He *et al*., 2017). The important lesson to be derived from these observations is that a newly invading arbovirus that finds immunologically naïve human populations can establish during interepidemic periods in tropical areas with entrenched populations of *Ae. aegypti*.
